# Age Dependent Changes in Cartilage Matrix, Subchondral Bone Mass, and Estradiol Levels in Blood Serum, in Naturally Occurring Osteoarthritis in Guinea Pigs

**DOI:** 10.3390/ijms150813578

**Published:** 2014-08-05

**Authors:** Jin-Yin Yan, Fa-Ming Tian, Wen-Ya Wang, Ying Cheng, Hua-Fang Xu, Hui-Ping Song, Ying-Ze Zhang, Liu Zhang

**Affiliations:** 1 Department of Orthopedic Surgery, Hebei Medical University, Shijiazhuang 050017, China; E-Mails: bc3368@dlmu.edu.cn (J.-Y.Y.); zhangyingze55@gmail.com (Y.-Z.Z.); 2 Medical Research Center, Hebei United University, Tangshan 063000, China; E-Mail: tianfaming55@gmail.com; 3 Department of Pathology, School of Basic Medical Sciences, Hebei United University, Tangshan 063000, China; E-Mail: wangwenya35@gmail.com; 4 Department of Orthopedic Surgery, Affiliated Hospital of Hebei United University, Tangshan 063000, China; E-Mails: chengying66888@gmail.com (Y.C.); xu@xxuhuafang7788@gmail.com (H.-F.X.); chelun@dlmu.edu.cn (H.-P.S.)

**Keywords:** osteoarthritis, cartilage, subchondral bone, estradiol, electron microscope

## Abstract

The Dunkin Hartley (DH) guinea pig is a widely used naturally occurring osteoarthritis model. The aim of this study was to provide detailed evidence of age-related changes in articular cartilage, subchondral bone mineral density, and estradiol levels. We studied the female Dunkin Hartley guinea pigs at 1, 3, 6, 9, and 12 months of age (eight animals in each group). Histological analysis were used to identify degenerative cartilage and electron microscopy was performed to further observe the ultrastructure. Estradiol expression levels in serum were assessed, and matrix metalloproteinase 3 and glycosaminoglycan expression in cartilage was performed by immunohistochemistry. Bone mineral density of the tibia subchondral bone was measured using dual X-ray absorptiometry. Histological analysis showed that the degeneration of articular cartilage grew more severe with increasing age starting at 3 months, coupled with the loss of normal cells and an increase in degenerated cells. Serum estradiol levels increased with age from 1 to 6 months and thereafter remained stable from 6 to 12 months. Matrix metalloproteinase 3 expression in cartilage increased with age, but no significant difference was found in glycosaminoglycan expression between 1- and 3-month old animals. The bone mineral density of the tibia subchondral bone increased with age before reaching a stable value at 9 months of age. Age-related articular cartilage degeneration occurred in Dunkin Hartley guinea pigs beginning at 3 months of age, while no directly positive or negative correlation between osteoarthritis progression and estradiol serum level or subchondral bone mineral density was discovered.

## 1. Introduction

Osteoarthritis (OA) is a heterogeneous condition characterized by structural and functional degradation of affected synovial joints [1]. The pathology and mechanisms leading to cartilage destruction, with associated pain and impaired movement, have traditionally dominated the attention of researchers. It is widely believed that if the causes and progression of this disease were better understood, an opportunity for knowledge-based treatment would emerge. The earliest osteoarthritic changes in human OA cannot be investigated for ethical considerations concerning tissue sampling. As a consequence, numerous animal models have been developed, but most are secondary OA models using mechanical instability or chemical interventions to drive the disease [2,3,4,5,6], which are not ideal for researching primary OA. The Dunkin Hartley (DH) strain of guinea pigs are one of the most widely used strains of spontaneous OA animal models since the appearance of joint pathology in both DH guinea pigs and humans is age-related [2,7]. Furthermore, the histological and biochemical changes in guinea pigs resemble that of human OA, and DH guinea pigs are subject to a variety of well-recognized OA risk factors that are shared with humans [8,9,10]. Articular cartilage changes in DH guinea pigs have been described using histology [11] electron microscopy [12] and molecular biology [13] separately; however, most of these previous studies focused on only one or a few of these changes, it is necessary to investigate all of these changes during entire OA progression in DH guinea pigs model.

Estrogen may have pro- and anti-inflammatory properties depending on the context and the tissue involved. In general, acute loss of estrogen increases the levels of reactive oxygen species and activates nuclear factor-κB and pro-inflammatory cytokine production, which is indicative of their predominant anti-inflammatory properties [14]. In clinical practice, the prevalence of OA is higher among women than men, and this prevalence increases considerably after menopause [14]. In addition, deletion of estrogen receptors in female mice [15] results in cartilage damage, osteophytosis, and changes in the subchondral bone of the joints, suggesting that estrogens have a protective role in the maintenance of joint homeostasis. It is necessary to clarify the changes in estradiol levels over the entire course of OA progression in guinea pig.

Therefore, the present study aimed to (1) describe the age-related changes in articular cartilage macroscopic and microscopic structure, the subchondral bone mineral density (BMD) and the glycosaminoglycans (GAG) and matrix metalloproteinase-3 (MMP-3) expression in cartilage in knee joints of DH guinea pigs; and (2) to investigate whether osteoarthritic cartilage change degeneration is related to changes in serum estradiol levels in this primary OA model.

## 2. Results

### 2.1. Gross Morphology and Histopathology Changes in Cartilage

**Figure 1:**
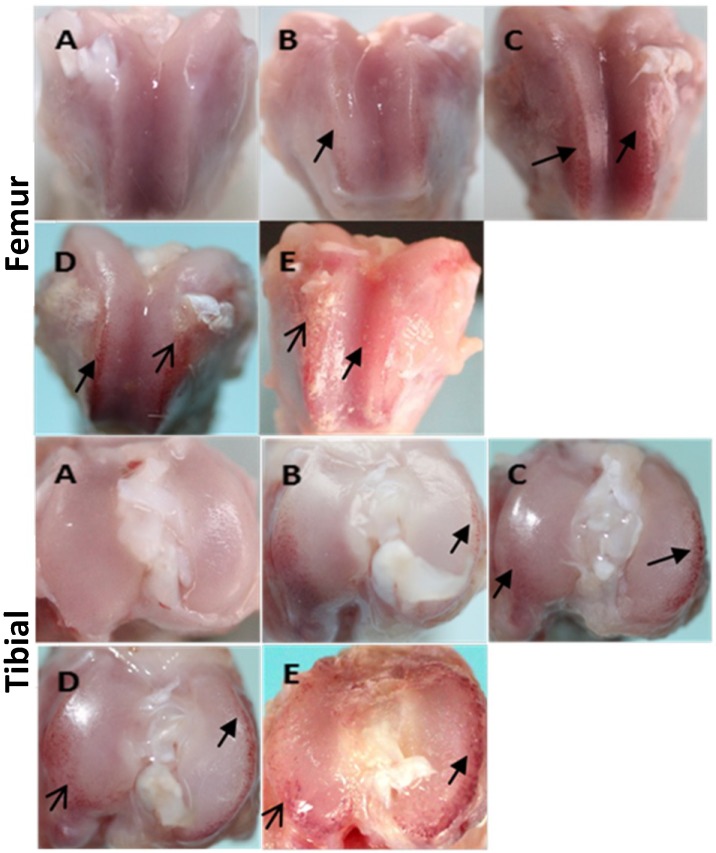
Outline of the distal femur and proximal tibial. (**A**) 1-month group; (**B**) 3-month group; (**C**) 6-month group; (**D**) 9-month group; and (**E**) 12-month group. Black solid arrow indicates ulceration, black arrow indicates osteophyte.

The articular cartilage in 1-month old animals was smooth and without any evidence of degeneration. At 3 months of age, mild ulceration occasionally appeared. By 6 months of age, cartilage ulcerations and matrix loss were obvious and osteophytes had begun to develop when animals reached 9 months of age; all of these features increased in severity by 12 months of age (Figure 1).

**Figure 2:**
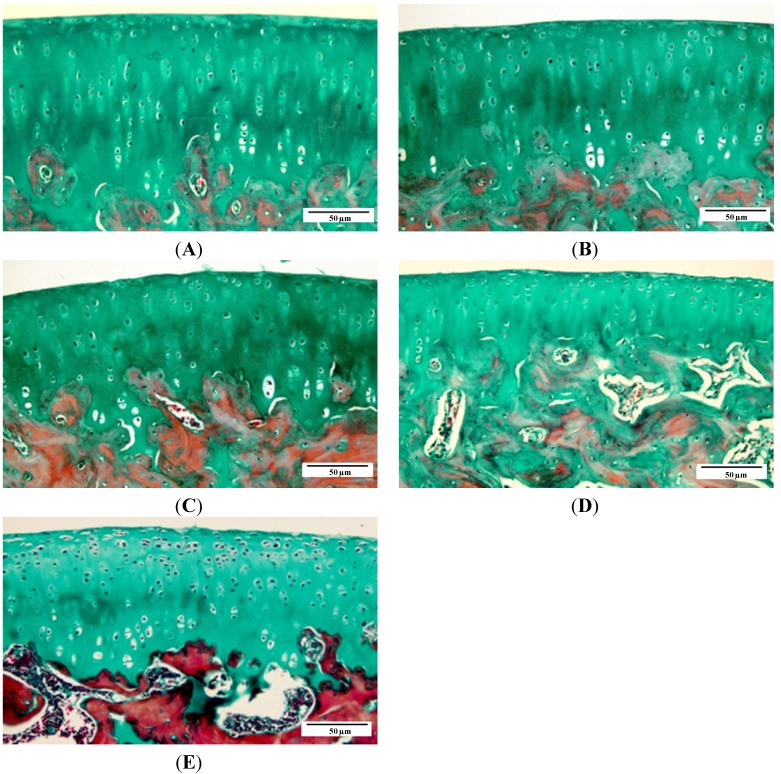
Histological findings of articular cartilage at distal femur. Representative sections are shown for each (×200). (**A**) 1-month group; (**B**) 3-month group; (**C**) 6-month group; (**D**) 9-month group; and (**E**) 12-month group.

As seen by Masson’s trichrome staining, articular cartilage from femoral condyle in 1-month old animals had normal cellularity and extracellular matrix. Early histological changes were observed in 3-month old animals, including focal proteoglycan loss and fibrillation. At 6 months old, animals displayed chondrocyte hypertrophy, “cloning” and surface cartilage lesions, and more obvious chondrocyte death/loss. Fibrillation and proteoglycan loss were observed in 9-month old animals, and erosions were shown in 12-month old animals (Figure 2).

**Figure 3:**
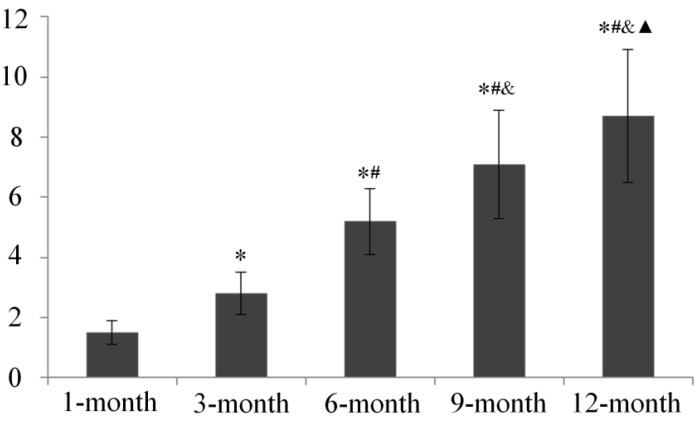
Mankin score of the articular cartilage in each age group. * *p* < 0.05, compared with 1-month group; ^#^ *p* < 0.05, compared with 3-month group; ^&^ *p* < 0.05, compared with 6- month group; ^▲^ *p* < 0.05, compared with 9-month group.

All of the aforementioned changes were semi-quantitatively confirmed by age-dependent increased histological score according to the Mankin scoring system (Figure 3).

### 2.2. Electron Microscopy Findings

**Figure 4:**
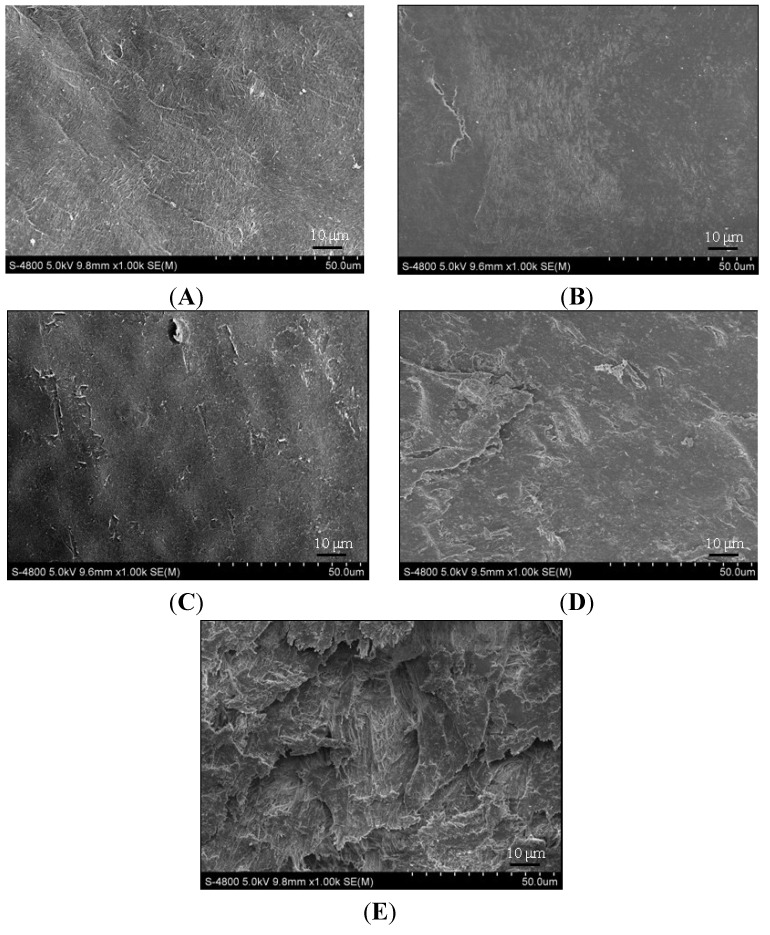
Findings of articular cartilage at proximal tibia under scanning electron microscope (SEM). (**A**–**E**) samples from 1, 3, 6, 9, 12 months old group respectively.

With respect to scanning electron microscope (SEM) observations, normal cartilage with a dense surface was shown in 1-month old animals; formation of micro-cilia was observed in some 3-month old animals; rough surface and even ulcerations were detected in 6-month old animals; and collagenous fibers degenerated into thick bundles and cracks emerged in 9-month old animals, which progressed to severe in 12-month old animals (Figure 4).

**Figure 5:**
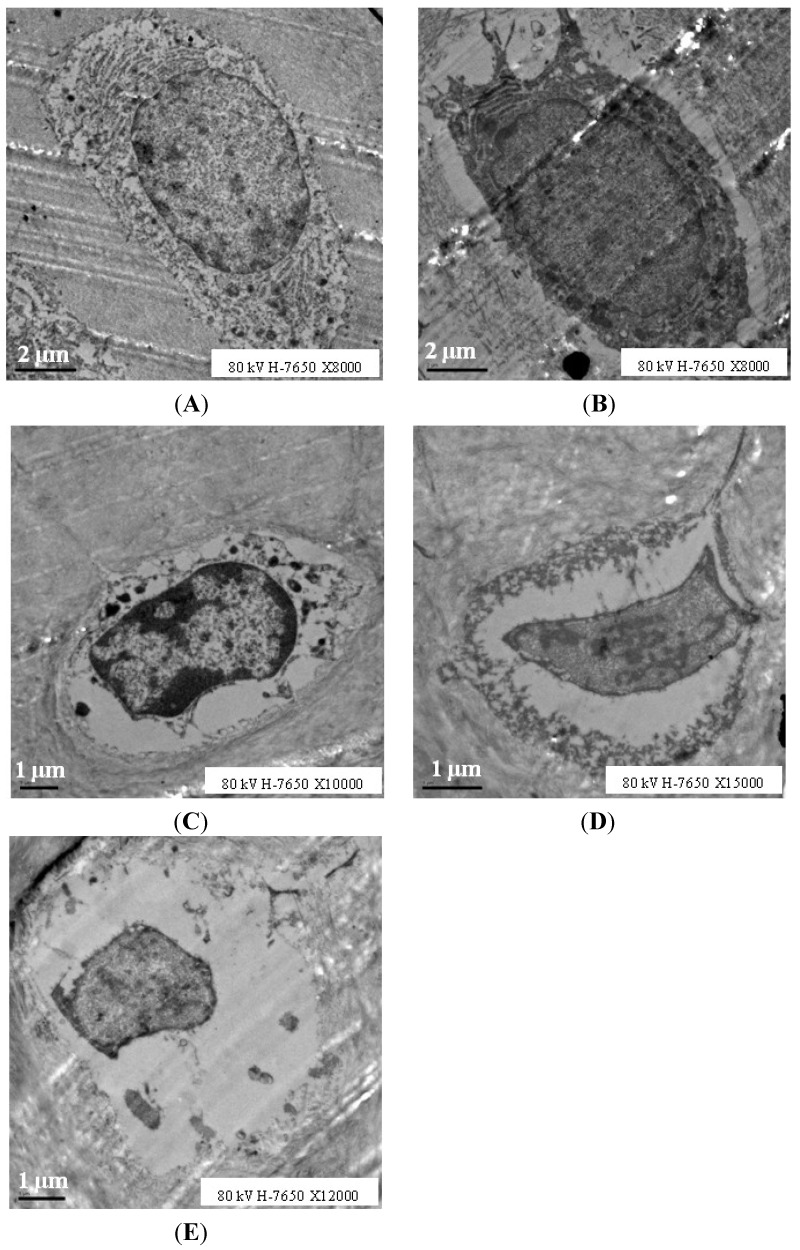
Transmission electron microscopy (TEM) findings of chondrocytes in proximal tibia articular cartilage. (**A**–**E)**, samples from 1, 3, 6, 9, 12 months old group respectively.

**Figure 6:**
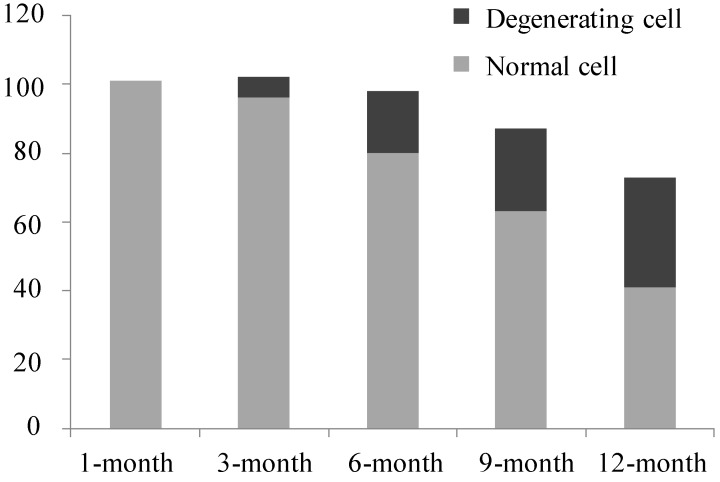
Number of normal and degenerating chondrocyte in each age group.

Transmission electron microscopy (TEM) revealed more details regarding cellular degenerations during OA progression in this model: More chondrocytes were observed in 1-month old animals and no apoptotic or necrotic chondrocytes were found; cell nuclei and membranes were integrated and rich in cytoplasmic organoids, chromatin was distributed uniformity, and few abnormal chondrocytes were detected in 3-month old animals, with irregular shapes and loss of cytoplasmic organoids. Age-related increases in apoptotic or necrotic chondrocytes were detected, as shown in Figure 5, the cavity around the cell became smaller with cellular and nuclear contraction, cytoplasmic organoid loss or disappearance, which we considered as degenerating cells according to the study from Ou *et al.* [16] and loss of normal chondrocytes occurred. For each age group, normal and degenerating cell numbers were calculated, as shown in Figure 6, and the percentage of degenerating cells increased in an age-related manner (Figure 5 and Figure 6).

### 2.3. Serum Estradiol Levels

**Figure 7:**
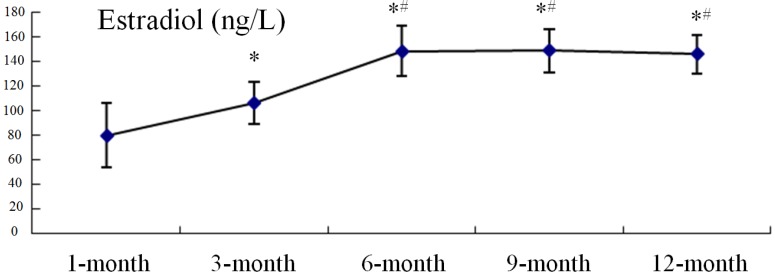
The serum levels of estradiol in guinea pigs with different ages.* *p* < 0.05, *vs*. 1-month group; ^#^ *p* < 0.05, *vs*. 3-month group.

From months 1 to 6, serum estradiol levels increased markedly, before remaining stable with no significant differences between any two groups (6-, 9-, or and 12-month old animals; Figure 7).

### 2.4. Qualitative Analysis of Matrix Metalloproteinase-3 (MMP-3) and Glycosaminoglycans (GAG) Expression

**Figure 8:**
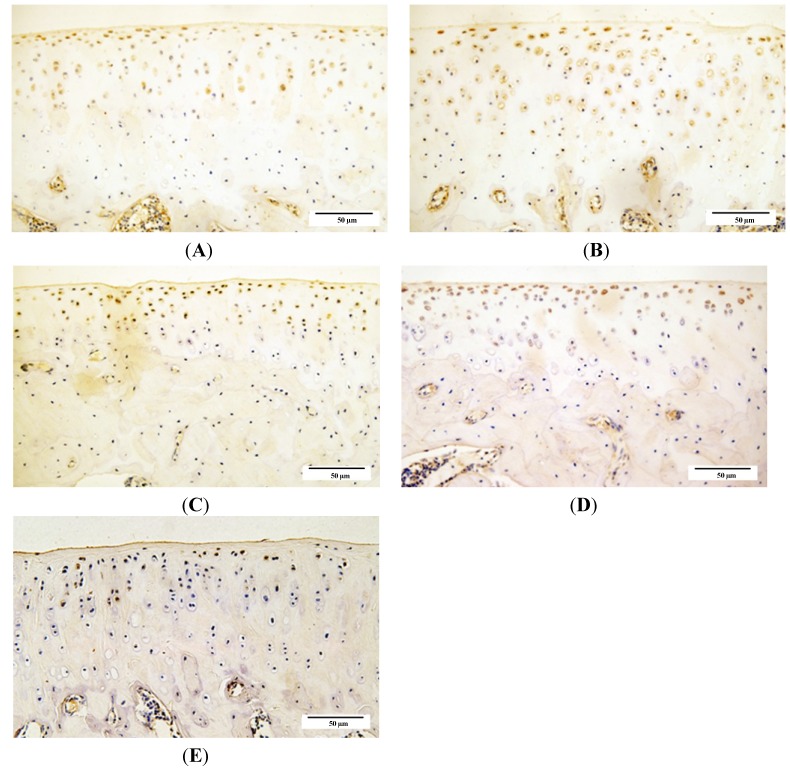
Immunohistochemistry assay for glycosaminoglycans (GAG) in each age group. (**A**) 1-month group; **(B**) 3-month group; (**C**) 6-month group; (**D**) 9-month group; and (**E**) 12-month group.

**Figure 9:**
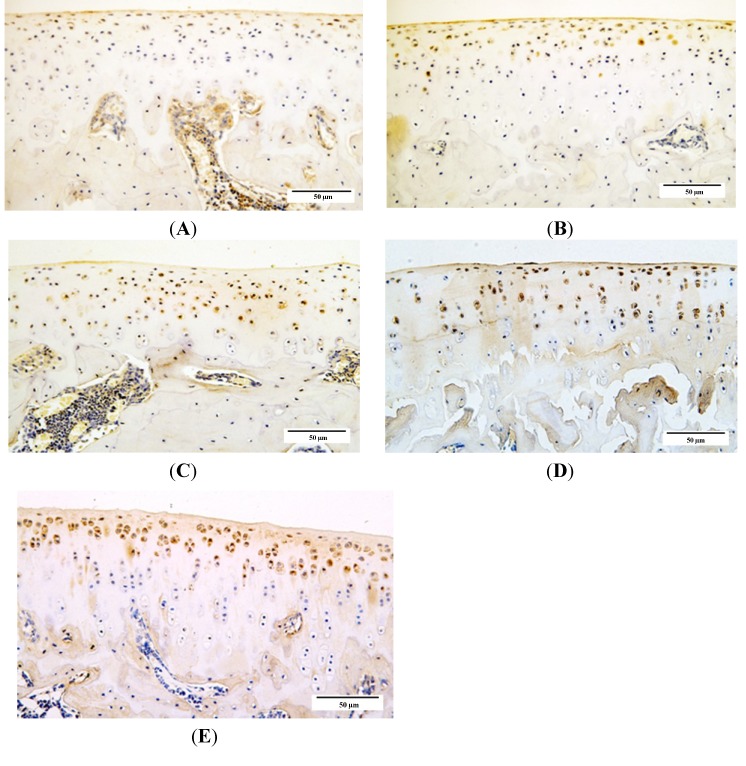
Immunohistochemistry assay for and matrix metalloproteinase-3 **(**MMP-3) in each age group. (**A**) 1-month group; (**B**) 3-month group; (**C**) 6-month group; (**D**) 9-month group; and (**E**) 12-month group.

**Figure 10:**
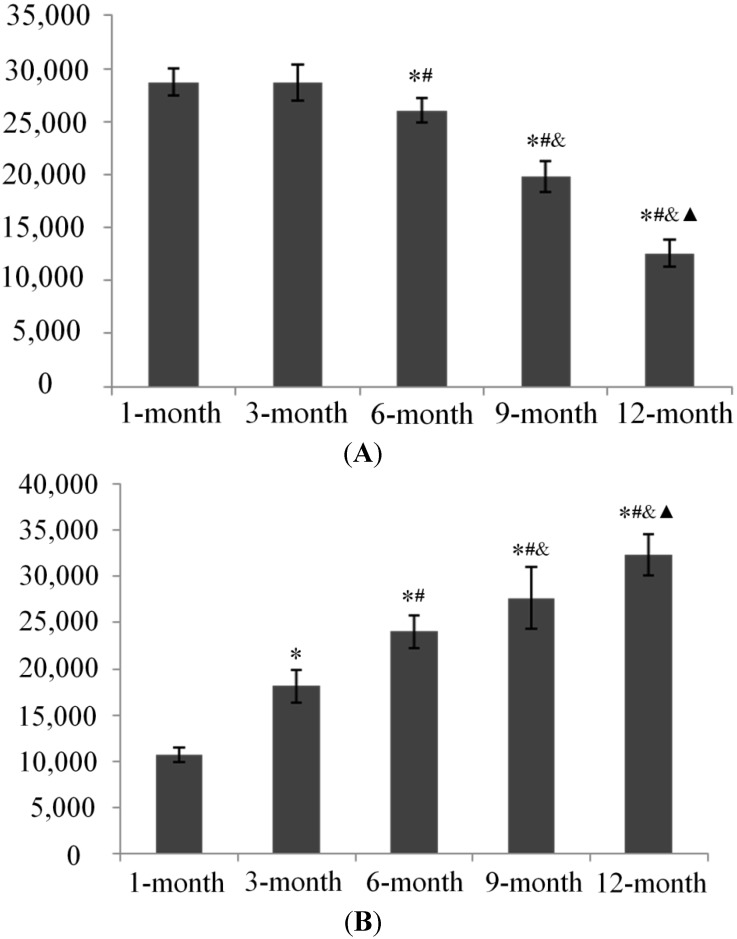
The integrated optical density (IOD) of GAG (**A**) and MMP-3 (**B**). * *p* < 0.05, compared with 1-month group; ^#^ *p* < 0.05, compared with 3-month group; ^&^ *p* < 0.05, compared with 6-month group; ^▲^ *p* < 0.05, compared with 9-month group.

We used immunohistochemistry to examine the distribution of matrix metalloproteinase-3 (MMP-3) and glycosaminoglycans GAG expression in knee joint cartilage from guinea pigs from each age group. The integrated optical density (IOD) values in each group showed that expression of GAG was intense in younger animals, with a significant age-related decrease in GAG expression beginning from 6 months of age. In contrast, an increase in MMP-3 expression was detected with increasing age of the animals (Figure 8, Figure 9 and Figure 10).

### 2.5. Subchondral Bone Mineral Density (BMD) Evaluation

**Figure 11:**
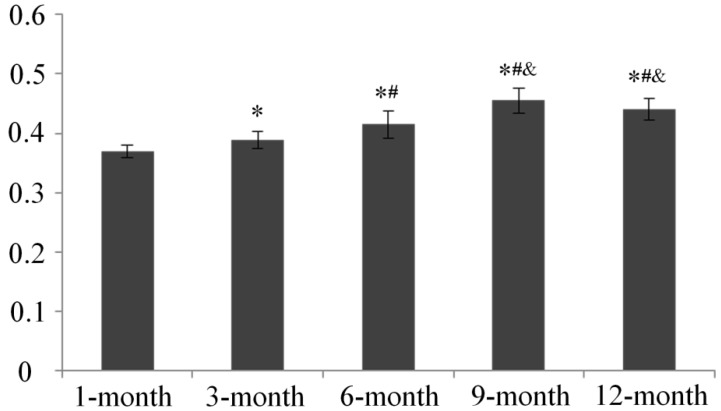
Bone Mineral Density (BMD) of medial proximal tibia in each age group. * *p* < 0.05, compared with 1-month group; ^#^ *p* < 0.05, compared with 3-month group; ^&^ *p* < 0.05, compared with 6-month group.

Bone mineral density (BMD) results showed age dependent increases in medial tibia subchondral bone mineral density from 1 to 9 months of age, with peak values at 9 months, and thereafter remained stable with no significant difference between this time point and 12-month old animals (Figure 11).

## 3. Discussion

The present study delivers a number of important findings by using histology, electron microscopy, immunohistochemistry, and serology techniques to analyze the complete progression of osteoarthritis (OA) in Dunkin Hartley (DH) guinea pigs. Though previous studies have reported some of these findings separately, most focus on a specific time period of this OA model. In our study, however, we used the same batch of animal at different ages and characterized healthy cartilage and mild, moderate, and severe articular cartilage degeneration, which provided detailed results regarding OA progression.

Histological analysis and scanning electron microscope (SEM) findings in our study suggested that significant age-related articular cartilage degeneration occurs in DH guinea pigs starting from 3 months of age, which is supported by other studies of spontaneous OA in DH guinea pigs [17,18]. Furthermore, increased chondrocyte apoptosis was reported to be associated with OA progression in spontaneous guinea pig models of this disease [19]. The transmission electron microscopy (TEM) results of our study suggest an age-related decrease in cell density and an increase in cartilage chondrocyte apoptosis and necrosis, of which cell apoptosis appeared earlier than cell necrosis. These findings provide direct evidence that the histological changes observed by Masson trichrome stain in this study, cellular changes in particular, are mainly caused by chondrocyte death, which is either dominated by apoptosis during the middle stage of OA or by necrosis in older animals with severe OA.

Cartilage degradation involves a variety of degradative enzymes, notably matrix metalloproteinase (MMPs) [20,21]. MMP-3 as one of the members in MMPs is involved in proenzyme posttranslational activation [21] and appears to be one of the few genes that is up-regulated during the early stages of OA [22]. Our data show that age-related increases in MMP-3 activity coincides with osteoarthritic changes in DH guinea pigs, which suggests that MMP-3 participates in the degradation of cartilage matrix during OA development as a catabolic metabolism stimulator. One of the changes that occurs during OA development is the loss of matrix proteoglycans [23]. Numerous studies suggest that most of the biological activity of proteoglycans is mediated by their GAG chains. Loss of proteoglycan GAG chains is an early event in OA resulting in cartilage degradation [24,25,26]. Our data demonstrates that GAG expression in DH guinea pig cartilage decreases since 6 months old, which occurred later than when the articular cartilage degeneration were observed and when increased expression levels of MMP-3 were detected. These results may be because of matrix catabolic metabolism progression during OA development, in which the matrix degradation is triggered by, and therefore occurs after, the activation of inflammatory factors including MMPs; moreover, the compensation of impaired chondrocyte metabolism in younger animals may partially contribute to this result.

Our data demonstrates that estradiol levels in DH guinea pigs increase markedly from 1 to 6 months and then remain stable, which does not parallel OA development. Among the multiple physiopathological mechanisms involved in OA, those related to sex hormone control have been attracting much attention, in particular those involving estrogens [27]. The loss of articular cartilage integrity has been shown in several animal models with estrogen deficiency [28,29,30,31,32] and this pattern was supported by a study by Claassen *et al.* [33], who showed that estradiol could suppress the expression of some degradative proteinases in cultured osteoarthritic chondrocytes. Estrogen deficiency plays a relevant pathogenic role in OA, and estrogen deprivation might exert a double effect on normal cartilage: on the one hand, through the direct action of estrogen itself, and, on the other, through the associated increase in subchondral bone turnover [34]. From these studies, it is reasonable to conclude that estrogen is a protective hormone for OA cartilage; however, there is no study focusing on estradiol level changes in blood serum during OA development in DH guinea pigs. In our study, we tested these levels by ELISA, and our data show that, with the development of OA, estradiol levels increase rapidly during early growth and remained stable after the guinea pigs reached 6 months old; the trend of estradiol changes in these animals at different ages reflects neither a positive nor negative relationship between estradiol and OA progression. Further study, for example investigating the expression levels of the estradiol receptor in cartilage chondrocytes, may be needed to clarify whether/how estradiol participates in OA development.

Although subchondral bone changes constitute a characteristic change in OA on a structural level, little is known about the role of subchondral bone in the disease process. Experimental and clinical data support the hypothesis that both high and low bone mineral density (BMD) conditions, including osteoporosis, may induce osteoarthritis [35]. Recently, several studies have focused on the role of subchondral bone in both OA initiation and progression. For instance, it was shown that tibial subchondral (TS) BMD may play a role in OA progression and could be used as a predictor of knee osteoarthritis progression [36], as patients in the lowest quartile of baseline BMD experienced less joint space narrowing than those in the highest BMD quartile. Our findings in the present study are showing that TSBMD increases from 1 to 9 months of age in DH guinea pig and may indicate a positive correlation between TSBMD and OA. However, a recent study reported that both systemic (total hip or spine) and subchondral BMD are positively associated with increased cartilage thickness in subjects with radiographic OA, suggesting that BMD might play a protective role against cartilage loss in knee OA [37]. Additionally, 6 months of age is associated attainment of skeletal maturity in guinea pigs [38]. In this context, regarding the age of the animals used in the present study, age-related increases in TSBMD may be more the result of growth than a trigger for OA progression. A study analyzing the relationship between TSBMD and OA in animals of the same age would possibly clarify this controversy.

By using multiple assessment methods to determine age-related changes in articular cartilage, serum estradiol levels, and subchondral BMD during OA development in DH guinea pigs, the present investigation provides detailed evidence not only about histological degeneration, but also cellular and molecular changes in this widely used naturally occurring OA model. Moreover, no directly positive or negative correlation between OA progression and serum estradiol level or subchondral BMD was identified in this study. Therefore, the roles of estradiol and subchondral bone in the development of OA in DH guinea pigs require further study.

## 4. Methods

### 4.1. Animal Handling

All experiments were approved by Hebei United University Animal Care and Use Committee, Tangshan, China. 41-month old female DH guinea pigs (Vital River Experimental Animal Technical Co., Ltd., Beijing, China) were fed a standard rodent diet and housed in the Medical Research Animal Center (Tangshan, China). The bodyweights of all animals were recorded.

### 4.2. Gross Observations and Specimen Processing

Animals were randomly sacrificed at either 1, 3, 6, 9, or 12 months of age (eight animals at each time point). Blood samples were harvested before sacrifice for enzyme-linked immunosorbent assay (ELISA) analysis of estradiol concentration. After disarticulation, both femurs and tibias were cleaned and their gross visual appearance recorded with a digital camera (Canon 550D, Canon Inc., Tokyo, Japan). The right femurs were harvested for paraffin sections and immunohistochemistry was conducted after femurs were stained with Masson’s trichrome stain. The harvested tibias were prepared for BMD assessment and electron microscopy analysis.

### 4.3. Preparation and Cartilage Histopathological Analysis

All right femurs were fixed in 4% paraformaldehyde for 48 h and then decalcified with 15% ethylenediaminetetraacetic acid (EDTA)-Na_2_ (pH 7.4) at 4 °C for 6 weeks. The distal end of the femurs were dehydrated, embedded in paraffin, and cut into 5-μm thick sections, as per a standard protocol. Four sections from each sample were stained with Masson’s trichrome stain (Bioss Inc., Beijing, China). Next, three color digital images from each section were recorded by light microscopy (Olympus BX61, Olympus Corporation, Tokyo, Japan). Semi-quantitative histopathological analysis was established according to the Mankin score system, which ranges from 0 to 14 using four characteristics: structure (0–6), cellularity (0–3), matrix staining (0–4), and tidemark integrity (0–1), where 0 represents normal cartilage and >10 represents severe cartilage degeneration.

### 4.4. Electron Microscopy Analysis

The medial tibial plateau from right knees were isolated for scanning electron microscope (SEM; *n* = 4) and transmission electron microscopy (TEM; *n* = 4). For SEM, the samples were fixed in 2.5% glutaraldehyde at 4 °C for 24 h and then dehydrated using an increasingly concentrated series of ethanol solutions. After further dehydration with acetone and isoamyl acetate, specimens were dried with Hitachi critical point drying unit (Hitachi, HCP-2, Ibaraki, Japan). After coating with a layer of gold, all specimens were studied with SEM (Hitachi S-4800, Ibaraki, Japan). For TEM, all specimens were normally demineralization and fixed in 2.5% glutaraldehyde and 2.0% paraformaldehyde for 4 h with 0.1 mol/L phosphate buffer at 4 °C. After fixation with 2% osmium tetroxide for 30 min, tissues were dehydrated in a series of graded ethanol solutions. Subsequently, ethanol was substituted with propylene oxide and then specimens were embedded in Epon 812 (MarSys-Tech, Beijing, China). Ultrathin sections (70 nm) were prepared and double-stained using uranyl and lead acetate before they were observed using TEM (H-7650, Hitachi Ltd., Tokyo, Japan); normal and degenerating cells were counted.

### 4.5. ELISA Analysis

The estradiol ELISA immunoassay kit (Xinle, Shanghai, China) was used to quantify the amount of estradiol in guinea pig blood serum. The estradiol assay was performed according to the manufacturer’s instructions. The optical density was measured at a wavelength of 450 nm using an ELISA microplate reader (iMark, Bio-Rad Laboratories Inc., Hercules, CA, USA); concentrations were calculated and are expressed as pg/mL.

### 4.6. Immunohistochemistry for Glycosaminoglycans and Matrix Metalloproteinase-3

To further clarify cartilage matrix metabolism during OA progression, glycosaminoglycans (GAG) and matrix metalloproteinase-3 **(**MMP-3) expression in cartilage were detected by immunohistochemistry. Briefly, tissue sections were routinely deparaffinized, rehydrated, and repaired in complex phosphoesterasum for 10 min at 37 °C, and then incubated overnight at 4 °C with anti-rabbit GAG (1:200; Boster Corporation, Wuhan, China) and MMP-3 (1:150; Boster Corporation, Wuhan, China) antibodies. The remaining procedures were adapted from the PV-6001 Two-Step IHC Detection Reagent illustration (ZSGB-BIO Corporation, Beijing, China); the brown color was developed using DAB (ZSGB-BIO Corporation, Beijing, China) and the sections were counterstained with hematoxylin (Baso Corporation, Zhuhai, China).

All sections were semi-quantitatively analyzed using Image Pro Plus (IPP; Media Cybernetics Inc., Rockville, MD, USA) version 6.0 software, and the integrated optical density (IOD) was measured from the images at 200× magnification.

### 4.7. Subchondral BMD Analysis

After muscles were removed, the BMD of the medial tibia plateaus was scanned using dual X-ray absorptometry (DXA) (QDR Discovery, Hologic, Bedford, MA, USA). The region of interest (ROI) was a 3 mm × 4 mm area located on the subchondral bone plate toward the epiphyseal plate [6]. Next, BMD in the ROI was measured using a small animal model resolution software program; measurements from eight knees per group were averaged.

### 4.8. Statistical Analysis

All data were analyzed using SPSS 17.0 software (IBM Corporation, Armonk, NY, USA) and results are expressed as means ± standard error of the mean (SEM). The Shapiro-Wilk test for normality and Bartlett test for homogeneity of variance were performed. Comparisons between groups were tested using a one way analysis of variance (ANOVA) and Fisher’s least significant difference (LSD) *t*-test. A *p*-value <0.05 was considered statistically significant.

## 5. Conclusions

Age-related articular cartilage degeneration occurred in Dunkin Hartley guinea pigs beginning at three months of age, in parallel with activate cellular degeneration and matrix catabolism, while no directly positive or negative correlation between osteoarthritis progression and estradiol serum level or subchondral bone mineral density was discovered.
